# The Freiburg sport therapy program for eating disorders: a randomized controlled trial

**DOI:** 10.1186/s40337-020-00309-0

**Published:** 2020-07-07

**Authors:** Almut Zeeck, Sabine Schlegel, Friederike Jagau, Claas Lahmann, Armin Hartmann

**Affiliations:** Department of Psychosomatic Medicine und Psychotherapy, Center for Mental Health, Faculty of Medicine, University of Freiburg, Hauptstraße 8, D-79104 Freiburg, Germany

**Keywords:** Exercise, Physical activity, Randomized trial, Anorexia nervosa, Bulimia nervosa, Sport therapy, Eating disorder, Exercise dependence, Compulsory exercise

## Abstract

**Background:**

Unhealthy attitudes towards sport and problematic exercise behavior in eating disorders (ED) are common and associated with poorer treatment outcome and higher relapse rates. There is a need to develop and empirically test interventions that could complement standard treatment. The study aimed to assess the efficacy of the Freiburg sport therapy program for eating disorders (FSTP).

**Methods:**

Outpatients with ED were randomized either to a 3 month sport therapy program (sport therapy group: STG) or a waiting list control group (CG). Patients were assessed when starting the program and at the end of the intervention. The intervention group (STG) was followed up after 6 month. Main outcome criterion was a reduction in unhealthy exercise (Commitment to Exercise Scale, CES). Secondary outcomes encompassed eating pathology (Eating Disorder Examination, EDE), different dimensions of unhealthy exercise (Compulsive Exercise Test, CET subscales) and exercise quantity (accelerometer).

**Results:**

Recruitment was challenging. Fifteen patients were randomized to the STG and 11 were randomized to the CG condition. There was no statistically significant difference between groups according to the main outcome criterion. However, the STG showed a significantly stronger reduction in avoidance and rule driven behavior (CET subscale) when compared to the CG. Improvements (STG) were maintained at follow up.

**Conclusions:**

There was no statistically significant difference in the reduction of unhealthy attitudes towards sport and problematic exercise behavior between the intervention and the group, as measured with the Commitment to Exercise Scale. Further findings may point to the effectiveness of the program, but have to be interpreted with caution and verified in further studies. A major limitation is the small sample size.

**Trial registration:**

Study register: ISRCTN 14776348 (registered 26 January, 2015.

## Plain English summary

Unhealthy attitudes towards sport and problematic physical activity behaviour are common among people with an eating disorder. They are associated with a worse course of the disease and higher relapse rates. There is a need to develop therapeutic approaches that address this problem area. Therefore, a sports therapy program was developed and evaluated in the present study. Fifteen patients participated in the sports therapy program and were compared with 11 patients who did not participate. All patients had an eating disorder and showed unhealthy attitudes towards sports and/or problematic exercise behaviour. Overall, fewer patients participated in the study than was intended. At the end of the program, the patients who participated in the sports therapy group felt less guilty and bad if they did not exercise. Other measures showed no differences between the groups. The improvements were maintained 6 months after the intervention. The results may indicate the effectiveness of the program, but must be interpreted with caution because of the small sample size and verified in further studies.

## Background

In general, exercise promotes physical and mental health [1]. However, in patients with eating disorders (ED), unhealthy forms of exercise (also referred to as compulsive exercise or excessive exercise) are a common phenomenon [2], yielding prevalence rates from 30% to over 80% in anorexia nervosa and from 20% to over 60% in bulimia nervosa [3]. The prevalence differs depending on the sample studied, the definition of unhealthy exercise and the measures used. Unhealthy exercising is regarded as a factor associated with poorer treatment outcome, e. g. higher relapse rates and a chronic course in anorexia nervosa, and longer hospital stays and a greater severity of eating pathology across ED diagnoses [4–6]. Although diagnostic criteria for anorexia and bulimia nervosa (DSM 5, ICD 10) refer primarily to the *quantity* of physical activity („excessive“), its *quality* can be considered more characteristic [7, 8]. Examples are exercising according to rigid rules, thinking obsessively about exercising, feeling guilty when missing exercise sessions, and continuing to exercise despite negative physical, psychological or social consequences [9].

The literature contains a multitude of terms (e.g. excessive exercise, compulsive exercise or exercise dependence), with no consensus on a definition. Some authors emphasize the rigid, obsessive and compulsive aspects of exercise (e.g. [4, 7, 10]), while others highlight the similarities to addictions [9]. Additionally, in some patients with anorexia nervosa and severe malnutrition, exercising may be driven partly by motor unrest and hyperactivity, which can be caused physiologically [11–13]. Due to the heterogeneity of the terms, we decided to use the comprehensive term “unhealthy exercise” in the following.

Unhealthy exercise in ED was found to be motivated by a range of factors [2, 14], the most important of which were to regulate tension or negative affect [15–18] and to influence weight and shape [14, 19–22]. Difficulties with the regulation of negative affect as well as concerns about weight and shape are main aspects of psychopathology in patients with ED [23, 24]. In a previous study using methods of ambulatory assessment in real life-situations, we could show that negative mood precedes exercise behavior in ED patients and that exercising was able to effectively influence mood as well as dysfunctional ED-cognitions subsequently, such as a drive for thinness and body dissatisfaction [25]. This was a temporary, short-term effect. It can be assumed that these effects contribute to the maintenance of unhealthy exercise behavior.

With regard to procedures in treatment, there is an ongoing debate on whether exercise in ED should be restricted versus included as a (supervised) therapy component [26, 27]. Although discussion about an appropriate handling of exercise in the treatment of eating disorders goes back a long way [28], the number of interventions that have been developed so far to specifically address unhealthy exercising is limited [3, 29]. Cook et al. [27] recently summarized the existing literature to derive recommendations for treatment strategies. Main components that were suggested by the authors are the following, if briefly summarized: Patients should be treated in a team of experts; written contracts will be helpful; the program should contain psychoeducation and a stepwise and individually adapted increase in physical activity intensity and a reflection on physical activity should be included.

Only few programs have been developed so far that entail most of the suggested components. They were either designed to be part of an inpatient program (see e.g. [30, 31] or were intended as an adjunct to outpatient psychotherapy (see for example [32–34]). The LEAP (Compulsive Exercise Activity Therapy) program for example, comprises 8 group sessions for outpatients. An adapted version was evaluated in a study that compared 34 individual sessions of cognitive-behavioral therapy for anorexia nervosa (AN) with 34 individual sessions of cognitive-behavioral therapy for anorexia nervosa in which 8 individual sessions oriented on LEAP were embedded [35]. Interventions led to improvements in eating pathology, BMI as well as in beliefs and attitudes towards exercise, but with no difference between treatments. Another program was evaluated in an observational study [30]. It was part of an inpatient treatment and group sessions were held four times per week. According to the progress of patients, the content of sessions changed - with an increase in intensity of exercise activities and autonomy. Patients who took part in the program showed a decrease in emotional commitment to exercise, in exercise involvement and exercise rigidity, while patients in a control group did not. The program did not adversely affects weight gain. However, results were difficult to interpret due to the observational design of the study. The same applies to a pilot study on HEB (Healthy Exercise Behavior), a group program (8 sessions, twice a week) that was also part of a multimodal inpatient treatment program. Again, positive changes (pre to post-intervention) were found concerning eating as well as unhealthy exercise [31].

Starting in 2008, we developed a sport therapy program for eating disorders that is intended to complement outpatient psychotherapy (“Freiburg Sport Therapy Program” FSTP, for details see below). The program was continuously improved, based on clinical experiences and interviews with patients [32]. Interviews with completers of the program revealed that patients experienced the group as a safe place and the therapist as a person, they could identify with and who stood for a healthy attitude towards physical activity. They described a better awareness of their physical limits, allowed themselves to reduce exercise quantity without feeling guilty, to have fun and to be less perfectionistic with exercises. However, patients who dropped out early described a fear of the group situation, a problem with rivalry with others or with getting in too close contact with their eating disorder and their body [32]. This led to a change of the content of the first session with a stronger focus on arriving in the group, reducing fears and conveying security.

The final version of the program encompasses 13 group sessions over a period of 3 months. In a pilot study including 18 patients, which was conducted between 2010 and 2012, we found a significant reduction of unhealthy exercise as measured with the Commitment to Exercise Scale (CES) as well as significant improvements in eating pathology and quality of life. The reduction of unhealthy exercise remained significant when compared to possible changes of unhealthy exercise in a matched control group, while changes in eating pathology and quality of life did not [32, 33]. We interpreted the findings in the direction that the program has specific effects on attitudes towards sports and exercise as well as exercise behavior.

**The aim of the recent study** was to evaluate the efficacy of the Freiburg Sport Therapy program in a randomized-controlled study. The target group were adult outpatients with an eating disorder that showed unhealthy attitudes towards sport and problematic exercise behavior. The main hypothesis was that unhealthy exercise (CES, total score) will be significantly more reduced in participants of the sport therapy program (STG) when compared to possible improvements in a waiting list control group (CG). Additionally, it was assumed that effects of the program will be maintained in a 6 months follow-up. Furthermore, we aimed to explore possible changes in eating pathology, different dimensions of unhealthy exercise and the quantity of physical activity.

## Methods

### Design

A randomized controlled study was conducted. After the recruitment of a sufficient number of patients for to start with a group cycle (> 10, at least 4 patients should be randomized into the intervention group), patients were randomized and allocated to the intervention (STG) or waiting list control group (CG). Randomization was conducted using a list of random numbers (www.random.org), generated by physical random processes.

CG patients were invited to join the next sport therapy group after 6 month to improve compliance with the study. However, this entailed the abandonment of a comparison of follow-up data.

The sport therapy program was conceptualized as an adjunct to outpatient treatment (individual psychotherapy). It was decided not to standardize outpatient psychotherapy, as most patients were already in treatment. A demand to stop a current treatment or change the psychotherapist was considered unethical and expected to led to problems in recruitment.

### Participants

Patients were screened in a specialized outpatient clinic for eating disorders at the Department of Psychosomatic Medicine and Psychotherapy (Center for Mental Health, Faculty of Medicine, University of Freiburg). Patients are usually sent to the outpatient clinic by psychotherapists in private practice for an additional assessment, or by their family doctors for a diagnosis, treatment recommendation and referral to psychotherapeutic treatment (outpatient, day hospital, inpatient unit).

As unhealthy exercise can be found in anorexia nervosa (AN) as well as in bulimia nervosa (BN) and there is considerable diagnostic overlap between diagnoses [3, 6, 23], it was decided to include both diagnoses in the sample. Unhealthy exercising was defined as having a CES total score above the Mean + 1 Standard Deviation of a healthy population that was recruited for a validation study (CES total score > 100) [36].

*Inclusion criteria* were a diagnosis of BN or AN according to DSM 5 (EDE interview by trained raters; one of the raters was a psychologist and one a sport scientist), an age ≥ 18 years*,* a score > 100 on the Commitment to Exercise Scale (CES) and a BMI ≥ 16 kg/m^2^ (to exclude patients with anorexia nervosa with a physical risk).

*Exclusion criteria* were being an elite athlete, being on a waiting list for inpatient admission, psychosis, substance dependency, organic brain disease and / or physical problems that did not allow participating in physical activities.

### Definition of drop out

Patients missing 5 consecutive group meetings were defined as drop outs and were excluded from the intervention group. Additionally, a weight loss of > 3 kg^1^ (since first assessment) or a BMI of < 15.0 kg/m^2^ was considered an adverse event (exclusion from further participation). All patients taking part in 2/3 or more of the group meetings were considered „completers“.

### Intervention

The Freiburg Sport Therapy Program was developed based on empirical evidence [2, 9, 29, 37] and practical experiences in a pilot phase [32]. The therapeutic stance was oriented on Calogero and Pedrotty [30], who emphasized the importance of a reflective exchange on participants thoughts and feelings related to practical experiences with physical activity and averted from a reward-punishment model. Aims of the program are the reduction of problematic attitudes towards exercise (“exercise quality”: e.g. exercising despite injury, feeling guilty when missing exercise sessions, exercising according to rigid rules, obsessive thoughts about exercise, exercising to influence weight and shape) and a change in problematic exercise behavior (“exercise quantity”) as well as enabling positive experiences with sport (socializing, fun, positive body experience, increase in self-efficacy). The manualized program was developed by a psychiatrist and eating disorder expert (AZ) and a sport-therapist and -scientist (SS). It comprises one introductory session plus 12 weekly group meetings (duration: 2 h) over a period of 3 months. It is a closed group with an optimal group size of 5–8 participants and led by a sport therapist with experience in working with eating disorders. The program is divided into 5 modules. These entail: A) psychoeducation, promoting a feeling of safety and cohesion in the group, B) self-monitoring (work with an exercise diary), observing and reflecting one’s own exercise behavior and attitudes, promoting body perception and getting to know healthy qualities of physical activity with simple exercises of lower intensity, C) working on behavior change in real-life situations, supporting healthy sport behavior, questioning a high degree of performance orientation in sports and working on perfectionism, stepwise increase in intensity, D) focus on playful and new experiences, trying out team games, transfer of group experiences into real life, E) reflection on experiences and changes made, plans for the future and saying good-by. Each group session started with a short round (e.g. what happened since last session) and had a strong focus on practical experience with physical activity and one’s own body. It closed with a final reflection round. Overall, the sport-therapist ensured an open climate in the group, made sure that the level of fear was not too high and that there was no overstraining pressure in the direction of change for to encourage new experiences with one’s own body and with exercise in the community with others.

### Assessment

STG patients were assessed at three time points of measurement: At randomization / start of the intervention (T0), at the end of the program (after 3 months, T1) and 6 months after the end of the intervention (follow-up, T2). Patients of the CG were assessed at T0 and T1 only. For a multi-level assessment, expert ratings as well as self-report instruments were used to evaluate eating pathology, general psychopathology and exercise behavior.

At T0, diagnoses were given after an EDE-interview (taking into account the diagnostic changes in DSM 5). All other measures were administered at each time point of measurement. Weight (BMI) was measured at T0, T1 and T2. In STG participants, weight was measured every 4 weeks for safety reasons, additionally.

*ED psychopathology* was assessed with the German version of the Eating Disorder Examination (EDE interview at T0, and the EDE- questionnaire (EDE-Q) at T0, T1 and T2; internal consistency: 0.80 ≤ Cronbach’s α ≤ 0.93 for factors and α = 0.97 for the total score) [38, 39]. The EDE encompasses four subscales: restraint (5 items), eating concern (5 items), weight concern (5 items) and shape concern (8 items). Thirteen additional items of the EDE interview allow a diagnostic assessment of the eating disorder according to DSM-5 and ICD-10. The items describe eating disorder-specific characteristics in their severity over the last 28 days. Higher scores indicate greater psychopathology. For most items, the frequency of a symptom is estimated, for seven items it is the intensity. The assessments are to be made on a seven-point rating scale (0 = not present to 6 = present on each day / in extreme severity). The total value is calculated from the sum of the scale mean values divided by the number of subscales.

Additionally, the Eating Disorder Inventory-2 (EDI-2, self-report; for factors: 0.82 ≤ Cronbach’s α ≤ 0.90) was administered [40–42]. Sixty-four items can be answered on a 6-point-Likert scale (0 = never; 5 = always). The EDI-2 contains 8 subscales, of which three were used in this study to describe the sample: drive for thinness, bulimia and body dissatisfaction.

*Unhealthy exercise* was measured with more than one instrument to cover different dimensions of the construct and to allow a comparison to other studies. As the main outcome measure the Commitment to Exercise Scale (CES) was used [10, 36]. It defines exercise as a “multifaceted construct” (entailing behavioral as well as attitudinal aspects of a commitment to exercise) and assesses compulsive and excessive features of exercise engagement that interfere with wellbeing, physical health and social responsibilities. The 8 items of the CES are answered on a visual analog scale (horizontal line of 155 mm) with bipolar items on each end (e. g. never versus always). The score for each item is constituted by the length of the line (from the beginning to the point marked). The validation of the German version revealed a one-factor solution [36] and showed a good internal consistency of the CES total score (Cronbach’s alpha = 0.82). The CES was able to differentiate between individuals with an eating disorder and individuals engaged in leisure time sporting activities [36]. Besides the CES, the Compulsive Exercise Test (CET) was administered [31, 43], which was more specifically designed to assess unhealthy exercise in ED and comprises the following subscales (24 items): Avoidance and rule driven behavior, weight control exercise, mood improvement, lack of exercise enjoyment and exercise rigidity (factors: 0.73 ≤ Cronbach’s α ≤ 0.88; total score: Cronbach’s α = 0.87). For each item patients were asked to rate how true the statement was on a Likert scale ranging from 0 = never true to 5 = always true. Furthermore, patients filled in the Exercise Dependence Scale (EDS), an instrument oriented on criteria of addiction [44–46]. The EDS has 21 items (each item is answered on a 5-point Likert-scale with the extremes never (1) and always (5)), a total score and the following 7 subscales: Withdrawal effects, continuance tolerance, lack of control, reduction of other activities, time and intention effects, as well as a total score (factors: 0.74 ≤ Cronbach’s α ≤ 0.95; total score: Cronbach’s α = 0.93). Only the total score was used in this study. For an assessment of the *quantity of physical activity*, patients wore an accelerometer attached to the right side of their hip (movisens Move-III) for 7 days before and after the intervention, during the time they are awake. An accelerometer measures physical activity objectively on the base of acceleration in a three-dimensional space (for detail see for example [47, 48]). Move-III comprises a triaxial acceleration sensor that measures movements with a sampling frequency of 64 Hz, a resolution of 12 bits and within a range of +/− 8 g. Finally, the International Physical Activity Questionnaire IPAQ [49] was used for self-assessment (Cronbach’s α not published, for a description of items and analysis see [49]).

*General psychopathology and depression* were assessed by the Global Severity Index of the Symptom-Check-List-27 (SCL-27 [50];; Cronbach’s α = 0.93), a short form of the SCL-90 (27 items describing possible symptoms a person can suffer from, are answered on a 5-point-Likert scale ranging from “not at all” (0) to “very strongly” (4)) and the Beck-Depression-Index (BDI-II [51]; Cronbach’s α = 0.88). The BDI-II encompasses 21 items, which can be answered on a 4-point Likert-scale (0–3) with four preset response options. The total score is obtained by adding the answers of all items.

*Primary Outcome* was defined as a reduction of the CES total score. Additionally, we aimed to explore possible changes in eating pathology (EDE-Q total score), in exercise quantity (accelerometer data), and in different dimensions of unhealthy exercise (CET-subscales) (secondary outcomes).

The patients could not be blinded with regard to the study condition. The blinding of research assistants conducting the initial assessment was possible in most of the cases.

Patients were asked for type, quality, frequency and duration of additional psychotherapy sessions received between T1 and T2.

### Sample size calculation

There were no comparable randomized controlled trials to base a power calculation on. For α = 0.05 and (1-ß) = 0.8 we determined the minimum sample size for an ANOVA for repeated measurement (time x treatment interaction). Power and Sample size calculations were computed with GPower V3.1. The total sample sizes to detect a significant difference were calculated to be *N* = 47 (CES) and *N* = 49 (EDE-Q). Given an expected attrition rate of 20%, we aimed to recruit 60 patients.

### Statistical analysis

The sample characteristics were obtained by simple descriptive statistics (means, standard deviations, frequencies, percentages). The randomization was checked by t-tests and crosstabulations (χ^2^-statistics) for independent samples. Change of scores were tested for significance with ANOVAs for repeated measurement. Two time points were available for the comparison of STG vs. CG; three time points were available for the STG. For the comparison of STG vs. CG a completer analyses (complete data, T0 → T1) as well as an intention to treat (ITT) analyses were conducted (missing data imputed by last value carried forward). Data analyses were carried out with SASJmp (V13).

In terms of accelerometer data, the calculation of energy expenditure and MET (metabolic equivalent of task) was done as follows (movisens algorithym): In a first step an activity class was estimated based on acceleration and barometric signals. Depending on the activity class, the corresponding model for energy expenditure/MET estimation was chosen. The model then takes the acceleration metric MovementAcceleration, the altitude change extracted from barometric data and the personal parameters age, gender, weight and height to calculate the energy expenditure and MET value. Energy expenditure/MET values are internally calculated for 1 min intervals independent of the configured output interval. MET is a measure of energy expenditure, which is defined as the ratio of metabolic rate during a specific physical task to a reference metabolic rate. Activity energy expenditure is the energy expenditure caused by physical activity, and total energy expenditure is the estimated total expenditure of a person. It is put together by the basal metabolic rate (BMR) and the activity energy expenditure. BMR is estimated by the WHO equations from age, gender, weight and height (see https://docs.movisens.com/Algorithms/energy_expenditure/#metabolic-equivalent-of-task-met).

## Results

### Participant flow and baseline characteristics

**Fig. 1:**
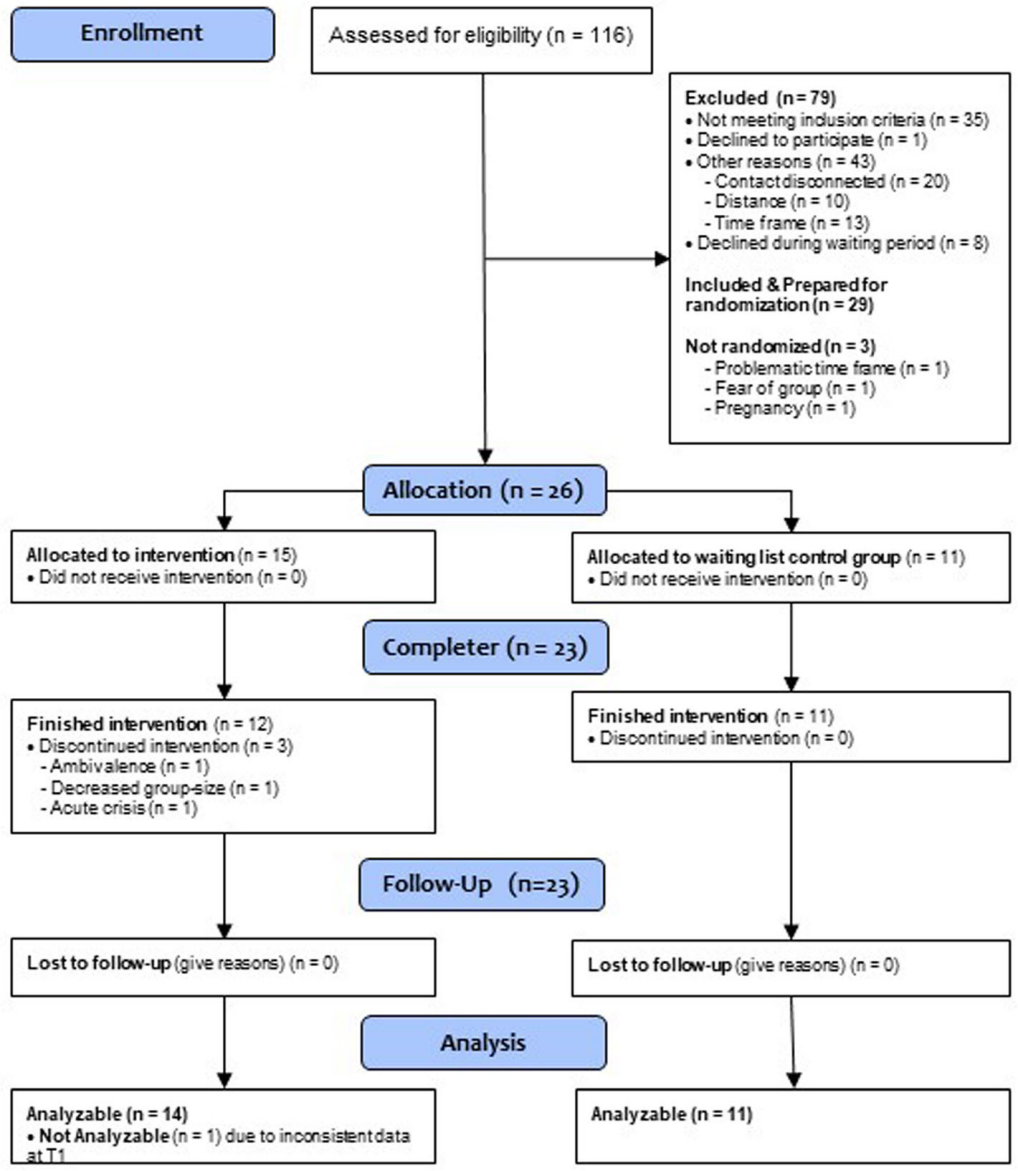
Flow chart

Despite the high prevalence rates of unhealthy exercise in ED patients, recruitment turned out to be a major challenge. Less than the intended number of patients could be included in the study (recruitment period: 1/2015–4/2018). The main reasons not to participate were a lack of motivation of many patients who did not want to change their exercise behavior and had a fear of the group situation (especially to be watched and a fear of rivalry and comparison). Furthermore, the long time span that was needed to recruit a sufficient sample size before randomization led to a situation in which patients lost their interest, started with a job that interfered with the group schedule or moved to another city. Overall, 26 patients could be randomized (for patient flow see Fig. 1). Patient characteristics at baseline are shown in Table 1.

**Table 1:** Sample characteristics at baseline

**Variable**	**STG (*N*** **= 15)** ***M (SD) / N (%)***	**CG (*N*** **= 11)** ***M (SD) / N (%)***	**t-test /Chi**^**2**^	***df***	***P***
**Sociodemographics**					
Age	24.3 (3.4)	27.2 (8.8)	1.17	24	0.25
Female	14 (93.3)	11 (100)	0.76	1	0.38
School > 10 years	15 (100)	9 (81.2)	2.96	1	0.09
BMI (kg/m^2^)	20.3 (2.7)	19.2 (2.1)	1.15	24	0.26
**Diagnosis**					
AN*	5 (33.3)	5 (45.5)	1.68	2	0.43
BN	9 (60.0)	4 (36.4)			
OSFED	1 (6.7)	2 (18.1)			
**Comorbidity** (axis I)					
Depression	4 (26.7)	2 (18.2)	0.25	1	0.61
Anxiety disorder	4 (26.7)	3 (27.3)	0.00	1	0.97
OCD	2 (13.3)	2 (18.2)	0.12	1	0.74
Other (dysthymia)	2 (13.3)	0 (0.0)	1.59	1	0.21
**Trt before randomization**^a^					
Current outpatient pt. (yes)	13 (93%)	11 (100%)	0.82	1	0.37
Number of sessions	21.9 (32.6)	55.6 (42.1)	2.0	18	0.06
**ED symptomatology**					
EDE interview total score	3.53 (1.19)	3.45 (1.13)	- 0.17	24	0.87
EDI-2 drive for thinness	11.87 (5.84)	11.5 (6.17)	- 0,15	23	0.88
EDI-2 bulimia	9.14 (6.21)	5.46 (3.98)	−1.71	23	0.10
EDI-2 body dissatisfaction	14.07 (6.52)	12.00 (8.73)	−0.68	23	0.50
**Exercise pathology**					
CES total score^a^	113.5 (14.0)	107.5 (24.5)	−0.77	23	0.45
CET total score^a^	17.4 (1.28)	16.9 (1.30)	−1.0	23	0.33
EDS total score^a^	74.4 (16.1)	68.4 (24.1)	−0.75	23	0.46
**SCL-27 GSI**^a^	1.92 (0.62)	1.13 (0.61)	0.43	23	0.67
**BDI-II total score**^a^	23.9 (9.05)	20.0 (7.46)	−1.14	23	0.27
**IPAQ**^a^					
Walking (MET)	1732 (1749)	656 (1623)	−1.82	24	0.08
Moderate (MET)	1421 (1463)	1287 (1276)	−0.24	24	0.81
Vigorous (MET)	3505 (2603)	3180 (2572)	−0.32	24	0.75
Sum (MET)	6659 (5233)	5123 (3798)	−2.54	24	0.42
**Accelerometer**^a^					
Step count^b^	102,514 (162799)	101,092 (38409)	0.063	22	0.86
Total energy expenditure^c^	14,420 (2324)	14,414 (1859)	0.007	22	0.59
Sedentary (1 - < 1,5 MET) ^c^	3947 (578)	4358 (903)	−1.363	22	0.19
Light (1.5 - < 3 MET) ^c^	1037 (322)	1077 (338)	−.298	22	0.79
Moderate (3–6 MET) ^c^	660 (315)	690 (338)	−.226	22	0.82
Vigorous (>6 MET) ^c^	152 (185)	199 (175)	−.625	22	0.54
Moderate to Vigorous	812 (481)	890 (444)	−.400	22	0.69
(≥3 MET) ^c^					
Weartime [h/day/participant]	13.88 (1.62)	15.23 (3.83)	1.193	22	0.25

Note: *Trt* treatment; *AN* anorexia nervosa (* STG: 93.3% restictive type; CG: 60% restictive type); *BN* bulimia nervosa; *EDNOS* eating disorder not otherwise classified; *EDE* Eating Disorder Examination (expert interview); *EDI* Eating Disorder Inventory; *CES* Commitment to Exercise Scale; *CET* Compulsive Exercise Test; *EDS* Exercise Dependence Scale; *IPAQ* International Physical Activity Questionnaire; *GSI* Global Severity Index, Symptom-Check-List 27; *BDI- II* Beck Depression Index; *MET* Metabolic Equivalent of Task

^a^ There were missing data from one patient

^b^ group-averaged sum of steps per participant across study week

^c^ group-averaged sum values per participant across study week

### Outpatient psychotherapy

The STG received 6.8 (*SD* = 2.8) psychotherapy sessions while in the program. In the CG, patients received 16.5 (*SD* = 2.5) sessions in the 3 month after the baseline assessment, showing a statistically significant difference (*T* = 2.59; *df* = 18; *p* < 0.02; missing data for *N* = 6).

### Attendance and acceptability of the program

Two patients did not start the program after randomization (one patient was highly ambivalent, another patient had an acute crisis) and one dropped out of the program after three sessions (overall drop-out rate: 3/15, 20%). This patient reported that the group situation was too difficult for her to handle, as the group was very small (4 participants) after the decision of another patient not to take part (see Fig. 1, “decreased group size”). None of the patients had to be excluded from the program because of safety reasons (e.g. weight loss).

### Primary and secondary outcomes (T0-T1)

Results are reported in Table 2. There was no difference between groups concerning the primary outcome criterion (CES total score). One statistically significant difference between STG and CG in a secondary outcome criterion was found: Avoidance and rule driven behavior was significantly more reduced after the sport therapy program compared to the CG in the intention to treat (ITT) as well as the completer analysis. There was no significant difference between groups in the reduction of overall eating psychopathology (EDE-Q total score) nor in the reduction of exercise quantity (accelerometer data).

**Table 2:** Group comparison at the end of treatment (sport therapy group vs. control group)

**Outcome**	**Group**	**Baseline (T0)** ***M (***±***SD)***	**End of intervention (T1)** ***M (***±***SD)***	**Group x time ITT-analysis** ***F (df)***	***p***	**Group x time Completer-analysis** ***F (df)***	***p***
**CES total score** (α = 0.66)°	STG	113.5 (13.97)	97.36 (24.24)	1.272 (1;23)	.271	1.773 (1;21)	.197
	CG	107.55 (24.48)	101.18 (24.77)				
**EDE-Q total score** (α = 0.92)	STG	3.84 (0.90)	3.66 (1.13)	0.008 (1;23)	.929	0.006 (1;21)	.937
	CG	3.41 (1.07)	3.20 (1.08)				
**CET-1 Av./ rule dr.** (α = 0.72)	STG	3.35 (0.42)	3.00 (0.58)	4.999 (1;23)	.035*	5.344 (1;21)	.031*
	CG	3.07 (0.92	3.18 (0.80)				
**CET-2 Weight contr.** (α = 0.59)°°	STG	4.03 (0.63)	3.94 (0.64)	0.014 (1;23)	.908	0.047 (1;21)	.830
	CG	3.80 (0.80)	3.75 (0.85)				
**CET-3 Mood impr.** (α = 0.73)	STG	4.23 (0.59)	4.19 (0.67)	0.364 (1;23)	.552	0.282 (1;21)	.601
	CG	4.49 (0.40)	4.29 (0.78)				
**CET-4 Lack enjoy.** (α = 0.57)°°	STG	1.78 (0.77)	1.57 (0.71)	0.299 (1;23)	.590	0.430 (1;21)	.519
	CG	1.30 (0.66)	1.21 (0.73)				
**CET- 5 Exerc. Rig.** (α = 0.50)°°	STG	3.90 (0.86)	3.54 (0.81)	0.235 (1;23)	.633	0.078 (1;21)	.783
	CG	4.27 (0.53)	3.76 (0.79)				
**CET-total score** (α = 0.53)°°	STG	17.29 (1.21)	16.24 (1.40)	0.158 (1;23)	.695	0.353 (1;21)	.559
	CG	16.93 (1.35)	16.18 (2.25)				
**EDS-total** (α = 0.94)	STG	70.93 (15.94)	67.50 (17.40)	0.995 (1;23)	.329	1.101 (1;21)	.306
	CG	74.36 (22.42)	75.18 (24.05)				
**Accelerometer**^a^							
Step count^b^	STG	102,514 (6279)	88,612 (61167)	0.014 (1;22)	.908	0.130 (1;18)	.986
	CG	101,092 (38409)	86,786 (37987)				
Resting Energy Expenditure (kcal) ^b^	STG	9151 (1309)	9157 (1461)	0.179 (1;22)	.677	1.546 (1;18)	.230
	CG	1321 (61)	1312 (74)				
Activity Energy Expenditure (kcal) ^b^	STG	5270 (1521)	4636 (1203)	0.506 (1;22)	.484	0.016 (1:18)	.900
	CG	5547 (1838)	4487 (1336)				
Total Energy Expenditure (kcal) ^b^	STG	14,420 (2324)	13,793 (1990)	1.049 (1;22)	.317	0.182 (1;18)	.675
	CG	14,414 (1859)	13,118 (1418)				
Sedentary (1- < 1.5 MET) ^c^	STG	3947 (578)	3600 (674)	0.669 (1;22)	.422	0.815 (1;18)	.379
	CG	4358 (903)	3692 (951)				
Light (1.5- < 3 MET) ^c^	STG	1037 (322)	894 (343)	0.227 (1;22)	.638	0.307 (1;18)	.586
	CG	1077 (338)	840 (315)				
Moderate (3–6 MET) ^c^	STG	660 (315)	606 (370)	0.173 (1;22)	.682	0.243 (1;18)	.628
	CG	690 (338)	582 (382)				
Vigorous (> 6 MET) ^c^	STG	152 (185)	119 (147)	0.004 (1;22)	.951	0.019 (1;18)	.891
	CG	199 (175)	158 (144)				
Moderate to vigorous (≥3 MET) ^c^	STG	812 (481)	725 (487)	0.143	.709	0.227 (1;18)	.639
	CG	890 (444)	740 (451)				

Note: *STG* sport therapy group; *CG* control group; *MET* Metabolic Equivalent of Task; *ITT* Intention-To-Treat Analysis

° = For the main outcome variable the obtained Cronbach’s alpha of the scale at T0 is artificially low, probably due to the inclusion criterion of a CES total score of > 100, restricting the possible range of scores and items; °° Cronbach’s alphas might be low due to heterogeneity of the constructs and a low number of items (*N* = 3) for two subscale (CET 4, CET 5)

^a^ IG: Missing data for one patients for T1 and T2 and missing data from a further patient for T2; CG: Missing data from two patients for T2

^b^ group-averaged sum of steps per participant across study week

^c^ group-averaged sum values per participant across study week

*Av./rule dr.* avoidance and rule driven behavior; *weight contr.* weight control; *mood impr*. mood improvement; *lack enjoy.* lack of exercise enjoyment; *exerc. Rig*. exercise rigidity

### Possible confounding variables

The following variables were planned to be analyzed as possible confounders: Depression severity (BDI-2), bulimic features (EDI-II, bulimia subscale) and number of psychotherapy sessions between T0 and T1. Unfortunately, the study ended with a considerably smaller sample size than planned and it now is underpowered for complex multivariate analysis. All confounder analyses yielded insignificant results. However, due to the lack of statistical power, this cannot be safely interpreted, neither for nor against confounders.

### Sustainability of results

For all outcomes, results in the STG were maintained at follow up or there was further improvement (see Table 3). There was continuous and significant improvement over time (T0 → T1 → T2) on all measures of exercise pathology (CES, EDS, CET). In particular, there was regard to eating psychopathology, there was no significant change from T0 to T1, but patients improved significantly in the follow up period.

**Table 3:** Follow-up, sport therapy group (STG, completer)

**Variable**	***N*** **= 12** ***M*** (***SD***)			**T0 → T1 → T2** ***F*** (***df)***; ***p***	**T0 → T1** ***F*** (***df)***; ***p***	**T1 → T2** ***F*** (***df)***; ***p***	**T0 → T2** ***F*** (***df)***; ***p***
	**T0**	**T1**	**T2**				
**CES total score**	112.58 (14.48)	94.00 (24.26)	80.33 (22.40)	2.02 (2;10); 0.004**	−2.65 (11); 0.02*	−1.57 (11); 0.16	−4.37 (11); 0.001**
**EDE-Q total score**	3.79 (0.96)	3.56 (1.19)	2.60 (0.89)	1.49 (2;10); 0.01**	−1.02 (11); 0.33	−2.84 (11); 0.015*	−4.04 (11); 0.002**
**EDS total score**	67.83 (14.89)	63.83 (15.80)	60.83 (17.52)	0.80 (2;10); 0.05	−1.22 (11); 0.25	−0.92 (11); 0.38	−2.97 (11); 0.013**
**CET total score**	17.36 (1.26)	16.12 (1.45)	16.09 (1.76)	1.82 (2;10); 0.006 **	−2.29 (11); 0.04*	−1.68 (11); 0.12	−4.38 (11); 0.001**
**CET – Av./rule dr.**	3.34 (0.49)	2.94 (0.61)	2.66 (0.58)	1.84 (2;10); 0.005**			
**CET – weight contr.**	4.03 (0.66)	3.92 (0.66)	3.58 (0.79)	0.38 (2;10); 0.20			
**CET – mood impr.**	4.18 (0.59)	4.13 (0.68)	4.15 (0.53)	0.01 (2;10); 0.96			
**CET – lack enjoy.**	1.91 (0.70)	1.66 (0.67)	1.41 (0.96)	0.49 (2;10); 0.14			
**CET – exerc. Rig.**	3.89 (0.93)	3.47 (0.85)	3.27 (0.72)	0.71 (2;10); 0.67			

Note: T2 is not available for the control group. Values of Table 3 are showing the development of scores in the intervention group only

*T0* baseline; *T1* end of intervention (after 3 month); *T2* follow-up (6 month after the end of intervention)

*Av./rule dr.* avoidance and rule driven behavior; *weight contr.* weight control; *mood impr.* mood improvement; *lack enjoy.* lack of exercise enjoyment; *exerc. Rig.* exercise rigidity

The one aspect of exercise pathology that significantly changed over all assessment points was avoidance and rule driven behavior (CET subscale), while exercising for mood improvement was the aspect that remained unchanged.

## Discussion

We evaluated the effects of the Freiburg sport therapy program in comparison to a waiting list control group in a randomized trial. There was no difference between groups with regard to a reduction in unhealthy exercise as measured with the Compulsive Exercise Scale (primary outcome). However, there was a significant change in „avoidance and rule driven behavior “ that was moderated by group (secondary outcome criterion). The subscale „avoidance and rule driven behavior “of the Compulsive Exercise Test (CET) comprises items that ask for negative affective consequences of missed exercise sessions (e.g. feeling depressed, guilty, irritated, angry) and the continuance of exercise despite being ill or injured [43]. These are criteria that can be considered at the core of unhealthy exercise in ED patients and are close to criteria for exercise dependence (withdrawal symptoms, continuance, [44]). An additional finding was an increase in variance in the total CES score at the end of the intervention, which may indicate that the intervention was effective in some patients and not in others. Unfortunately, the sample size was too small for a subgroup analysis that would allow the groups to be distinguished.

The follow-up assessment showed continuous improvement on all measures of unhealthy exercise, from the initial assessment to 6 month after the intervention. It is unlikely that this can be explained by spontaneous change and one might interpret these results – although with a lot of caution - as a hint for the effectiveness of the program. However, our design did not allow a comparison with the control group 6 months after the intervention.

In terms of further secondary outcomes, we did not find a difference between groups with regard to a change in overall eating pathology and a change in exercise quantity. Since the sport therapy program did not specifically address ED pathology, the intervention will, if at all, only have indirect effects on ED pathology through changes in exercise quality and quantity. Therefore, we did not really expect a significant difference. Indirect effects on ED pathology can be assumed, for example, through a more positive perception of the body, positive interactional experiences and an increase in self-efficacy. Interestingly, eating psychopathology showed a significant reduction in the follow up period. However, it cannot be derived from the data, if this change is a time-delayed effect of the intervention itself.

Furthermore, there was no significant difference between groups with regard to changes in exercise quantity. In descriptive terms, a slight reduction in all parameters was found in both groups. Unfortunately, we cannot compare this finding with data from other intervention studies, as these did not include an objective measurement of physical activity [30, 31, 35, 52]. In sum, the intervention did not induce much change here. Since we have no data on physical parameters like body composition, it is not possible to say how many patients showed a level of physical activity that was a danger to their health. The average amount of exercise in our sample in terms of steps and moderate or vigorous physical activity at baseline was considerably high when compared to young, female, healthy controls (for a comparison group see supplement in [25]), but with a wide range of values. Some patients showed an amount of physical activity that was clearly beyond the level that can be considered healthy. However, in other patients, exercise quality was the bigger problem than quantity. These patients reported an agonizing occupation with thoughts about exercise and physical activity, which was in contrast to their actual behavior. This is in line with previous findings and clinical reports [7]. Therefore we think that both aspects - exercise quality and quantity - should be focused on in a specialized sport therapy intervention. However, our findings make it necessary to discuss whether the intervention in the recent format sufficiently addresses and supervises the actual quantity of exercise behavior.

Finally, one further finding should be mentioned: If one considers possible changes in the attitudes towards exercise and exercise behavior as assessed with the CET (baseline to 6 months post intervention), one subscale showed no reduction at all: The subscale that measures exercising for mood improvement. It is a subscale that in another study showed at best minimal differences between patients with an eating disorder and a healthy control group (see [25]). This finding fits to research results showing that exercise in ED is often used to regulate difficult emotions [53]. We would like to question the view that this is always problematic. Our program aimed at changing pathological attitudes and motives related to exercise and at promoting its healthy use. In terms of healthy use, physical activity could be a possible coping strategy for ED patients when feeling tense or depressed – if exercising is used flexibly and in a non-compulsive manner.

Comparing the characteristics of our sample to that of other studies, patients had much higher scores for unhealthy exercise (CET, CES-Scores), due to the inclusion criteria, but similar values for eating pathology (EDE) [35, 52]. Our group program was intended to induce change in a group that showed significant pathology in the area of exercise. Therefore, we did not include patients without unhealthy exercising. At the end of the intervention, scores for exercise pathology were reduced, but they were still higher as the baseline-scores in most other studies. It has to be discussed and evaluated in further trials, if an intervention with the duration of 3 month is enough to induce sufficient and long-lasting change.

When patients started with the program, it was well accepted in nearly all of the cases. Only one patient dropped out. However, it was difficult to get patients motivated to participate in the group, although therapists knew about the typical fears. On the basis of these experiences, it should be discussed whether a sport therapy intervention should better be integrated into a multimodal program, in which it is an integral and obligatory part.

Overall, we think that conducting an outpatient group program for patients with an eating disorder that has a focus on the body and physical activity is challenging, but can be a helpful supplement to individual psychotherapy. The Freiburg sport therapy program was led by a sport therapist who had experiences in working with groups in sports and in working with eating disorders. Additionally, there was the possibility of supervision – for example in the case of difficult group dynamics. Further studies have to show if the manualized program can easily be taken over by other therapists or group leaders (e. g. physiotherapists or sport teachers). We think these should at least have experiences in working with sport and movement exercises as well as in working with groups of eating disordered patients.

Strength of the study comprise the randomised-controlled design, the inclusion criteria (patients with unhealty exercise only) and the multilevel measurement, including an objective assessment of physical activity. However, there are major limitations. Above all, the small sample size has to be mentioned. Some of our (non-significant) results might be due to the small sample size resulting in a lack of statistical power. We experienced profound difficulties with recruitment in an outpatient setting. Despite great efforts we could recruit only 48.3% (prepared for randomization *N* = 29) of the planned sample of 60 randomized cases. The block randomization design required recruiting simultaneously for the STG and CG, resulting in long waiting times for the first recruited patients, who dropped out for a range of reasons especially if their motivation to participate was ambivalent. The clinical experience with this study suggests that integrating such an exercise intervention into an inpatient or day hospital program might be more advantageous, as patients can be reached more easily. A further limitation of the study is the dispense to standardize outpatient psychotherapy (patients got “outpatient treatment as usual”, on which we have limited information), which might have influenced our results. Furthermore, there was a significantly higher number of sessions in the control group and we do not know if exercise was addressed in the psychotherapy sessions. A further limitation is related to the design: We offered patients of the CG to join the next sport therapy group after 6 month. As follow-up data were only available for the intervention group, results have to be interpreted with caution: It is not known whether the maintenance of effects is due to the intervention itself or to other influences, e.g. regular therapy, in the follow-up period.

## Conclusions

There was no statistically significant difference in the reduction of unhealthy attitudes towards sport and problematic exercise behavior between the intervention and the group, as measured with the Commitment to Exercise Scale. Further findings may point to the effectiveness of the program, but have to be interpreted with caution and verified in further studies. A major limitation is the small sample size.

## Data Availability

The datasets used and/or analysed during the current study are available from the corresponding author on reasonable request.

## Abbreviations

AN: Anorexia nervosa
BDI: Beck Depression Index
BMI: Body Mass Index
BN: Bulimia nervosa
CES: Commitment to Exercise Scale
CET: Compulsive Exercise Test
CG: Control group
ED: Eating disorder
EDE: Eating Disorder Examination
EDE-Q: Eating Disorder Examination – Questionnaire
EDI: Eating Disorder Inventory
EDS: Exercise Dependence Scale
HEB: Healthy Exercise Behavior
IPAQ: International Physical Activity Questionnaire
ITT: Intention to treat
LEAP: Compulsive Exercise Activity Therapy
SCL: Symptom Check List
STG: sport therapy group program

## Footnotes

1 A weight loss of > 20% in 6 months is considered an indication for inpatient treatment in the German S3-guideline for the treatment of eating disorders (https://www.awmf.org/leitlinien/detail/ll/051-026.html) ; this corresponds to a weight loss of about 3 kg in a patient with a BMI of 17 kg/m^2^ in a period of 1.5–2 months
